# Post-Receptor Inhibitors of the GHR-JAK2-STAT Pathway in the Growth Hormone Signal Transduction

**DOI:** 10.3390/ijms19071843

**Published:** 2018-06-22

**Authors:** Maciej Wójcik, Agata Krawczyńska, Hanna Antushevich, Andrzej Przemysław Herman

**Affiliations:** The Kielanowski Institute of Animal Physiology and Nutrition, Polish Academy of Sciences, ul. Instytucka 3, 05-110 Jabłonna, Poland; a.krawczynska@ifzz.pl (A.K.); a.antuszewicz@ifzz.pl (H.A.); a.herman@ifzz.pl (A.P.H.)

**Keywords:** growth hormone receptor, STAT, SOCS, protein tyrosine phosphatase, SIRT, SHP, PIAS, SIRPα1

## Abstract

The growth hormone (GH) plays a key role in the regulation of metabolic processes in an organism. Determination of the correct structure and functioning of the growth hormone receptor (GHR) allowed for a more detailed research of its post-receptor regulators, which substantially influences its signal transduction. This review is focused on the description of the post-receptor inhibitors of the GHR-JAK2-STAT pathway, which is one of the most important pathways in the transduction of the somatotropic axis signal. The aim of this review is the short characterization of the main post-receptor inhibitors, such as: cytokine-inducible SH2-containing protein (CIS), Suppressors of Cytokine Signaling (SOCS) 1, 2 and 3, sirtuin 1 (SIRT1), protein inhibitors of activated STAT (PIAS) 1, 3 and PIAS4, protein tyrosine phosphatases (PTP) 1B and H1, Src homology 2 (SH2) domain containing protein tyrosine phosphatase (SHP) 1, 2 and signal regulatory protein (SIRP) α1. The equilibrium between these regulators activity and inhibition is of special concern because, as many studies showed, even slight imbalance may disrupt the GH activity causing serious diseases. The regulation of the described inhibitors expression and activity may be a point of interest for pharmaceutical industry.

## 1. Introduction

### 1.1. Somatotropic Axis

Growth hormone (GH), one of the major hormones of the somatotropic axis, is a 191-amino acid protein found in many isoforms [1]. The human GH gene family is composed by two GH genes (*GH1* and *GH2*) and three placental lactogens (*CSH1*; *CSH2*; *CSHL1*) that are located in the chromosome 17 [2,3]. In adults the main expression of *GH1* is stated in the anterior part of pituitary, which is responsible for the action of this hormone at the endocrine level, but it is found also in numerous tissues and cells, in which GH acts in an auto/paracrine manner [4]. The regulation of the somatotropic axis activity is very complex. The basic pattern of regulation is connected with the feedbacks between hypothalamic neurohormones (growth hormone releasing hormone (GHRH) and somatostatin (SST)), GH and insulin-like growth factor 1 (IGF-1) [5]. However, lately, more and more factors regulating the activity of this axis have been discovered, such as ghrelin [6,7,8], leptin [9,10], cortistatin, [11] or klotho [12]. Many factors inhibit GH-induced hepatic expression of IGF-1, therefore inhibiting the IGF-1 effect on hypothalamic somatostatin release and the direct negative effect of IGF-1 on pituitary somatotrophs, thus acting as a modulator of GH impact on body growth or other IGF-1 dependent GH effects on the human body [13,14].

The role of GH in organism is mostly related to body growth and development. However, the range of actions of GH is pleiotropic. GH participates in the regulation of, for example, metabolism, immunology and reproduction. Both inhibition and enhancement of the GH activity may be harmful for the organism. Consequences of its deficiency include short stature, decreased bone mineral density and concentration, decreased muscle strength, thin skin and hair, delayed puberty and increased adiposity and hepatic steatosis along with impaired cognitive ability in several fields [15,16]. On the other hand, the excess level of this hormone causes, e.g., gigantism, cardiomyopathy, hypertension, arrhythmias, heart failure, diabetes, osteopenia, hypogonadism, thyroid goiter, proximal myopathy, polyps of colon or visceromegaly [17]. In addition to GH, the effects of somatotropic axis activity are exerted also by IGF-1. IGF-1 is a very potent growth factor which stimulates to growth all cell types [18] playing a key role in pre- and postnatal growth. It also directly affects glucose and protein metabolism [19]. During postnatal period of life, endogenous and exogenous IGF-1 was shown to promote the regeneration of different tissues, including the bone [20], muscle [21], nerve [22,23] and pancreas [24,25]. These pro-regenerative effects of IGF-1 are additionally associated with reduction in the release of pro-inflammatory cytokines and stimulation of the release of anti-inflammatory cytokines [24]. GH and IGF-1 may also participate in the therapeutic effect of other molecules such as ghrelin in pancreas [26], colon [27,28] or oral mucosa [29].

### 1.2. GHR-JAK2-STAT Pathway

**Figure 1:**
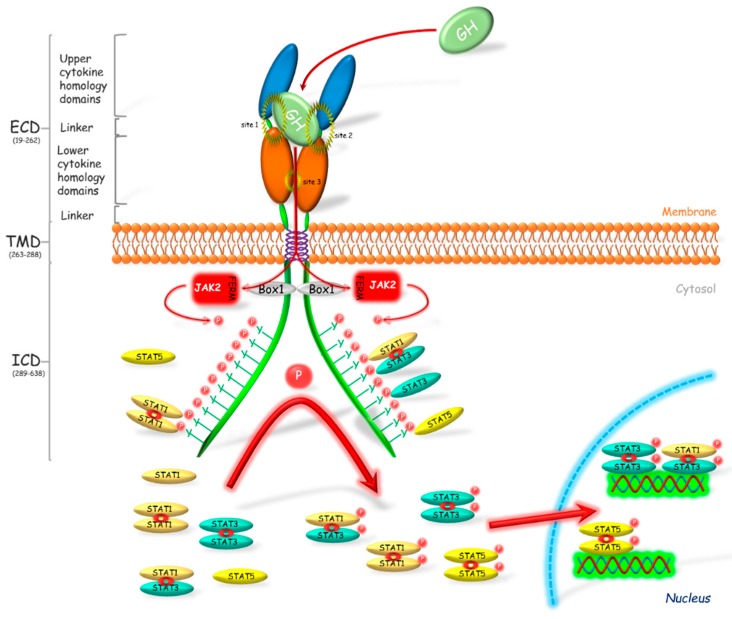
Activation of the growth hormone receptor and its signal transduction. Growth hormone (GH) binds to and activates its dimerized receptor (GHR). The activation leads to JAK2 binding via N-terminal 4.1, Ezrin, Radixin, Moesin (FERM) domain to Box 1 motif of the GHR and subsequently to phosphorylation of the intracellular tyrosine residues. The phosphotyrosines provide sites for binding of the various target signaling proteins including STATs, which in consequence leads to their phosphorylation. STATs are present in the cellular cytoplasm mostly in dimerized form. After phosphorylation STATs translocate into the nucleus where these transcriptional activators bind to appropriate promoter regions on DNA, which results in the transcription of gene or a set of genes. Box 1—proline-rich domain; ECD—extracellular domain; FERM—N-terminal 4.1, Ezrin, Radixin, Moesin domain; ICD—intracellular domain; JAK2—Janus kinase 2; P—phosphorylation marker; STAT—signal transducer and activator of transcription; TMD—transmembrane domain.

Growth hormone receptor (GHR) has been classified as a class 1 signaling molecule since the crystal structure of GHR-GH complex was revealed as 2:1 ratio between the receptor and hormone [30] (Figure 1). It was considered that the bounding of GH to its receptor caused the receptor dimerization and activation of associated signaling pathways ([31], one of the last reviews describing the wrong manner of the receptor activation). However, Gent et al. [32] showed that both GHR and other cytokine receptors such as prolactin, erythropoietin or thrombopoietin receptors, exist also as a dimer in the unbound with ligand form. Thus, GH binds to GHR dimer, which modifies position of the GHR extracellular domains and causes the activation of associated tyrosine kinases and therefore transduction of signal [33]. The changes that occur during receptor activation were widely described by [33,34].

The GHR is responsible for regulation of many processes including erythropoiesis, myelopoiesis, lactation, growth and metabolism. This is a single membrane pass receptor with the characteristic structure of the extracellular domain built of two fibronectin III-like modules. These modules with seven stranded β-sandwich structure are the centers of ligands binding. The common element of this class of receptors is the Box 1 motif which is present on the intracellular domain. Box 1 plays a crucial role in the transduction of the GHR signal. Binding of GH to its receptor causes a rapid binding of Janus kinase 2 (JAK2) to Box 1 via the N-terminal 4.1, Ezrin, Radixin, Moesin (FERM) domain [35] and the phosphorylation of JAK tyrosines which then phosphorylate many target proteins [36]. JAK participates in the activation of most pathways associated with GHR and plays a key role in signal transduction of the somatotropic axis. GHR transduces its signal mostly by the JAK2-signal transducer and activator of transcription (STAT) pathway [15]. Alves dos Santos et al. [37] observed that JAK2 forms a complex with GHR after the receptor internalization, which suggests that GHR is active also at the endosomal level. The GHR activation and the subsequent JAK2 phosphorylation, forms the binding sites for Src homology 2 (SH2) domain, which is present, among others, in STAT1, 3 and 5 [38]. Binding of these proteins to JAK2 through the SH2 domain causes their phosphorylation. Further phosphorylation of STATs on the level of Ser residues follows after their translocation to the nucleus [38,39]. Wen et al. [40] stated that for the full activation of STAT3 phosphorylation of Tyr705 and Ser727 is required. Similarly, for the activation of STAT1, phosphorylation of Tyr701 and Ser727 is essential. In turn, in the activated STAT5α, Ser779 [41], Ser725 [42], Tyr694 [43] and phospho-serine site located in a conserved Pro-Ser-Pro motif 726 (PSP726) are phosphorylated. Moreover, it was stated that STAT5α has a place of the Ser780 phosphorylation with Ser-Pro motif (SP) [44]. In turn STAT5β is phosphorylated at locations Tyr699 [43,45] and PSP731. Moreover, Mitra et al. [46] showed that STAT5β is phosphorylated by a cytokine induction at Ser193, which plays a crucial role in its subsequent transcriptional activity.

STAT proteins are present in varied structural forms, e.g., monomers, homodimers and heterodimers (Figure 1). Initially it was believed that these proteins are present in the cytoplasm in monomeric form until phosphorylation, which causes their dimerization [47,48]. In vitro studies conducted on HepG2 and A375 cell lines by Haan et al. [49] showed that STAT1 and STAT3 form complexes before the phosphorylation. Authors suggested also that STAT1 and STAT3 may not occur in the free form at all. Ndubuisi et al. [50] observed that STAT3 occurs in the monomer form rarely and the majority is present in the form of high molecular mass complexes: 200–400 kDa statosome I and 1–2 MDa statosome II, while the molecular mass of a single STAT3 molecule is 91 kDa. The presence of STAT1 and STAT3 as dimers in the cytoplasm of “non-activated” cells was confirmed also by Braunstein et al. [51], whereas, in the case of STAT5 protein, there are no reports of polymerization without prior phosphorylation.

Among the STATs, STAT5β plays the most important role in the transduction of GH signal into a cell. Udy et al. [52], who conducted research on mice with knocked out gene encoding STAT5β, observed the substantial growth disturbances. Moreover, the aforementioned authors compared the impact of the lack of STAT5β between genders of animals and observed the growth disturbances in both sexes, but the effect was more pronounced in males. Also Teglund et al. [53], who conducted study on mice with knocked out genes encoding STAT5α and STAT5β, stated the disturbances in the concentration of the IGF-1. Authors observed decreased level of IGF-1 only in males which was mediated, as the authors suggest, by a decreased level of testosterone to the level found in females. Such results indicate a participation of the pathway associated with testosterone in growth differentiation in this species. This fact may partly explain changes of a phenotype in humans and animals suffering from acromegaly or GH deficiency [54]. Similarly human studies showed that STAT5β deficiency causes growth shortage. Kofoed et al. [55] described the case of a patient with a homozygous mutation in the *STAT5β* gene causing deficiency of IGF-1, which expression is strictly correlated with STAT5β [56]. In IGF-1 signaling, the important role is also played by acid labile subunit (ALS) and insulin growth factor binding protein 3 (IGFBP-3). Moreover, the expression of these proteins is also associated with the STAT5β [57,58].

The aim of this review is to present factors that can modulate the activity of the GHR-JAK2-STAT pathway which has the most pronounced role in the somatotropic axis signal transduction. The review is focused on the post-receptor regulators of GHR transduction pathway, which affect the axis in an inhibitory manner, such as cytokine-inducible SH2-containing protein (CIS), suppressor of cytokine signaling (SOCS) 1, SOCS2, SOCS3, sirtuin 1 (SIRT1), signal regulatory protein α1 (SIRPα1), protein inhibitor of activated STAT (PIAS), protein tyrosine phosphatase-1B (PTP-1B), PTP-H1, SH2 domain containing protein tyrosine phosphatase-1 (SHP-1) and SHP-2.

## 2. GHR-JAK2-STAT Inhibitors

### 2.1. Suppressors of Cytokine Signaling (SOCS)

SOCS are also known as STAT-induced STAT inhibitory proteins (SSI) and their expression is induced by an activation of the JAK-STAT signaling pathway. Hitherto, 8 members of the SOCS family have been described: CIS and SOCS1–7 [59]. The SOCS proteins family is characterized by a specific protein structure as all of them have a centrally located SH2 domain and the SOCS box domain located at C-terminus of protein [60]. SOCS box is an ubiquitination related domain associated with complexes of elongin C and B, cullin-5, RING-box and ligase E2 [61], so SOCS proteins may act as ubiquitin E3 ligands and degrade proteins by binding to their N-terminus [62]. It was shown that SOCS box is a key mediator of the GH-GHR-JAK2-STAT5β pathway signaling inhibition [63] which, as already mentioned, is the most important pathway mediating the effects of GH [64].

It was shown that four SOCS proteins participate in the GH signal inhibition: CIS and SOCS1–3. As it was stated in many studies, expression of aforementioned SOCS proteins are induced by the GH action [65,66,67], and each had an inhibitory effect on the somatotropic axis signal transduction mediated via GHR [65,68,69]. As it was demonstrated by Leroith and Nissley [64], SOCS2 is responsible, among others, for regulation of the IGF-1 expression in the liver which is mainly mediated by STAT5β. The studies conducted by Greenhalgh et al. [63] and Vidal et al. [70] also confirmed the presence of mechanism in which SOCS2 expression increased in response to GH signal resulting in binding of STAT5β to the SOCS2 promoter, and by this way the negative signaling loop is created. The same authors proposed also another mechanism of SOCS2 interaction in which SOCS2 decreases GHR activity by lowering JAK2 activation. The significance of the effect of SOCS2 on the regulation of GHR activity is illustrated by study of Metcalf et al. [71] conducted on mice with *SOCS2* gene deletion. Aforementioned team demonstrated that SOCS2 knock-out (KO) mice were significantly larger than wild-type (WT) mice. The difference in weight gain became significant after weaning and, moreover, was associated with significant increase in bone length and proportional increase in weight of most organs. Authors stated that the activity of the somatotropic axis deprived of SOCS2 regulation was enhanced, resulting in, among others, increased level of IGF-1 mRNA expression in heart, lungs or spleen. However, there was no significant increase of this gene expression in bones, adipose tissue, muscle and liver, which is quite surprising in the light of other researchers’ results [63,70].

CIS and SOCS1–3 proteins exhibit a varied mechanism of action, different pattern of expression and intensity of inhibitory effect in response to different cytokines and GH action. Adams et al. [65] conducting study on the preadipocyte cell line showed that the expression of SOCS3 increased suddenly and transiently in response to GH treatment. Their results were confirmed by Tollet-Egnell et al. [66] in research on the primary cultures of rat hepatocytes. The authors showed that the increase of SOCS3 mRNA expression was sudden and transient in manner, with a 6-fold increase following after 30 min after GH stimulation. In turn, in case of SOCS2 and CIS the pattern of gene expression was slower and more prolonged than observed for SOCS3. The expression of SOCS2 mRNA increased significantly after 30 min from GH treatment and grew throughout the experiment, reaching 20 times of its initial level after 24 h. In *CIS* gene expression the first peak of the mRNA level was observed after 60 min and later increased between 4th and 24th h after the GH administration reaching the peak of the 10 times of its initial value. The varied kinetics of SOCS proteins genes expression and so their different inhibitory pattern on GH signal transduction may be due to differences in their structure. SOCS1 and SOCS3, unlike SOCS2 and CIS, have a small kinase inhibitory region (KIR domain) located at N-terminus [72,73], which acts as a pseudosubstrate inhibiting JAK-STAT pathway [73].

**Figure 2:**
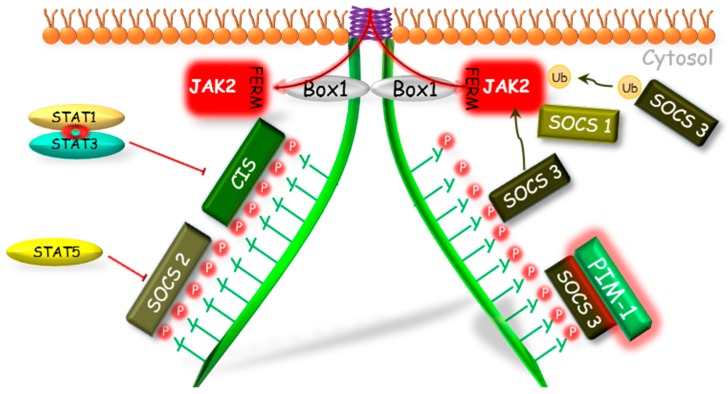
The SOCS proteins mechanisms of GHR-JAK2-STAT pathway inhibition. The SOCS1, 2, 3 and CIS have the same mechanism of phosphorylation inhibition of STAT by competitive binding to phosphotyrosines of the GHR. The SOCS1 and SOCS3, because of the KIR domain presence, can act also in a different manner. Both of them can bind to JAK2; however, SOCS1 can bind directly to JAK2 inhibiting its enzymatic activity and SOCS3 before binding to JAK2 requires binding to GHR, which leads to degradation of the GHR-JAK2 complex directly or indirectly by ubiquitination. Another mechanism of SOCS1 and SOCS3 action is dependent on the PIM-1 protein, which probably stabilizes their phosphorylation and prolongs their inhibitory action. CIS, SOCS—suppressors of cytokine signaling; Box 1—proline-rich domain; GHR—growth hormone receptor; JAK2—Janus kinase 2; FERM—N-terminal 4.1, Ezrin, Radixin, Moesin domain; KIR—kinase inhibitory region; P—phosphorylation marker; PIM-1—proto-oncogene serine/threonine-protein kinase; STAT—signal transducer and activator of transcription; Ub—ubiquitination.

In the regulation of the activity of GHR pathway, all engaged SOCS proteins (CIS, SOCS1–3) have the same mechanism of phosphorylation inhibition of STATs by the competitive binding to phosphotyrosines of the GHR (Figure 2). The SOCS1 and SOCS3 because of the KIR domain presence can act also in a different manner. Both of them can bind to JAK2 in bimodal way by two domains: N-terminal KIR domain which binds to catalytic groove of JH1 domain, and SH2 domain which binds to the phosphorylated Y1007, which is crucial in the activation loop of JH1 [72,73]. Hansen et al. [68] showed that only SOCS1 protein was able to inhibit the tyrosine phosphorylation in the conditions of JAK2 overexpression, while SOCS3 was able to inhibit the JAK2 tyrosines phosphorylation stimulated by GH only in the presence of the GHR. This indicates a varied mechanism of interaction of these proteins in GHR pathway and suggests that SOCS1 may bind directly to JAK2, whereas SOCS3 must be previously bound to GHR, which provides higher affinity [74]. As it was supposed by Wang et al. [75], SOCS3 may block the GH-dependent JAK-STAT signaling at various levels: by competitive inhibition of STAT5β phosphorylation or by binding to GHR, which leads to direct or indirect via elongin BC and GHR-JAK2 complex degradation and finally to the loss of JAK2 activity.

As it was mentioned above, CIS and SOCS2 also bind to receptor phosphotyrosine and in this case the mechanism of GH signaling inhibition is based on the competitive to STATs binding to the GHR complex [76]. The inhibitory effect of SOCS2 on GH signal transduction was firstly demonstrated by Favrea et al. [77]. As indicated by the authors, in previous studies on the SOCS2 inhibitory effect on the GH signaling [65], only high SOCS2 concentrations had been used, which did not affect GH in the inhibitory way. Favrea et al. [77] showed a dual nature of the SOCS2, which at low concentration acts as an inhibitor of the GH signal transduction, while at the high dose SOCS2 can restore the GH signaling by reducing the other SOCS proteins effects. The same authors confirmed the existence of an analogous negative regulation in the case of SOCS1. Subsequent studies indicate that at higher concentration SOCS2, by binding to GHR, may compete with more potent GH signal transduction inhibitors such as SHP-2 [78] or SOCS3 [68,69]. Tannahill et al. [79] demonstrated that SOCS2 significantly reduced SOCS3 protein expression in response to cytokines, while SOCS3 mRNA remained unchanged. The team found that SOCS2 could have affected the expression of SOCS3 protein in a proteasome-dependent manner and, consequently, enhanced the transduction of signal inhibited by other SOCS proteins. The results of Tannahill et al. [79] were confirmed by Piessevaux et al. [80] who shown that expression of SOCS2 completely suppressed the SOCS1 and SOCS3 dependent inhibition of GH signaling in HEK293-T cells. This interaction, as the authors claim, requires the presence of SOCS box in SOCS2 and recruitment of elongin BC complex, thereby supporting the proteasomal degradation of target SOCS proteins. Ram and Waxman [81] investigated the inhibitory mechanism of the CIS protein and its role in GH signal transduction inhibition via GHR-JAK2 complex under continuous exposure of the COS-1 cell culture on GH. It has been shown that CIS inhibits GHR-JAK2 activity by two different mechanisms: (1) the initial inhibition, in which a decreased GH-stimulated STAT5β level is stated due to CIS and STAT5β competitive binding to GHR phosphotyrosines or (2) the time-dependent inhibition connected with proteasomal degradation of CIS with accompanied degradation of GHR-JAK2 complex. Such time-dependent inhibition was not observed in case of SOCS1 and SOCS3. Besides that the SOCS3-dependent ubiquitination through SOCS box motif of the granulocyte colony-stimulating factor receptor was stated [82]. It was shown that in CIS and SOCS2 activity, ubiquitination may play more fundamental functional role as the SOCS1 and SOCS3-SOCS boxes were shown to bind Cullin5 with 100- and 10-fold lower affinity, respectively [83].

Moreover, several mechanisms modulating action of SOCS proteins have been discovered. Peltola et al. [84] demonstrated that a Proto-oncogene serine/threonine-protein kinase 1 (PIM-1) interacts with SOCS1 and SOCS3 and enhances their inhibitory effect on STAT5, most likely via SOCS phosphorylation stabilization. These results suggest that both PIM-1 and SOCS may participate in the mechanism of the negative loop which regulates the activity of STAT5.

### 2.2. Sirtuin 1 (SIRT1)

In mammals sirtuins are a family of nicotinamide adenine dinucleotide (NAD+) dependent enzymes that show homology to *Saccharomyces cerevisiae* gene silent information regulator 2 (Sir2). In humans, seven sirtuins (SIRT1–7) that regulate varied metabolic pathways have been found [85]. The most widely studied sirtuin is SIRT1, which plays a key role in the organization and stabilization of the genome, response to stress, glucose homeostasis or cell differentiation [86,87,88]. This protein also functions as a regulator in processes such as cell survival, inflammation, mitochondrial biogenesis and oxidative damage [89].

**Figure 3:**
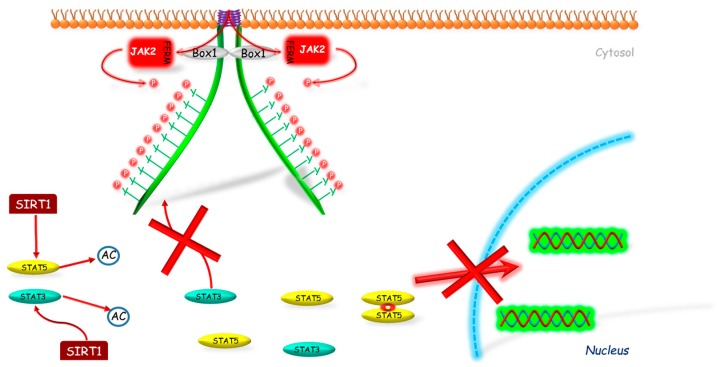
The mechanism of SIRT1 inhibition of JAK2-STAT pathway. SIRT1 interacts with STAT3 and/or STAT5 deacetylating these molecules, which prevents their further phosphorylation and by this way their translocation into the nucleus. AC—acetylation marker; Box 1—proline-rich domain; FERM—N-terminal 4.1, Ezrin, Radixin, Moesin domain; JAK2—Janus kinase 2; P—phosphorylation marker; SIRT1—sirtuin 1; STAT—signal transducer and activator of transcription; red cross—inhibition.

It was also found that SIRT1 can inhibit GH signaling. Yamamoto et al. [90] investigated the SIRT1 role in GH pathway signal transduction. Firstly, the authors showed that SIRT1 modulates GH action in the liver basing on the studies conducted on mice with knockdown SIRT1 protein in liver and on hypophysectomized mice. The negative impact of SIRT1 on GH-induced IGF-1 mRNA expression was also confirmed by the same team in HepG2 (human hepatocellular carcinoma cell line) and rat primary hepatocytes with use of SIRT1 inhibitors (sirtinol, nicotinamide) and stimulators (resveratrol, NAD). Continuing their research, Yamamoto et al. [90] showed that SIRT1 decreases Lys acetylation on STAT5 and inhibits the GH-induced Tyr phosphorylation on STAT5 (Figure 3). The same team investigated also the role of SIRT1 in GH resistance state. The authors stated that in fasted mice, SIRT1 protein level was increased; however SIRT1 inhibitor administration (nicotinamide) restored the Lys acetylation of STAT5 and STAT5 phosphorylation to the basic levels, which reversed the GH resistance state. On the other hand, in well-nourished conditions, treatment with SIRT1 stimulator (NAD) resembles the effect of fasting in terms of changes in GH signaling.

The inhibitory effect of SIRT1 was observed also on STAT3 protein activity. Nie et al. [91] conducted studies on immobilized mouse hepatocytes of SV40 cell line. The authors demonstrated that in cells treated with SIRT1 inhibitor (EX527) the level of acetylation and phosphorylation of STAT3 increased in a dose-dependent manner, and its activity was independent from JAK2. On the other hand, in cells treated with SIRT1 stimulator (resveratrol) the obtain effect was opposite. To determine if STAT3 deacetylation requires SIRT1, the authors conducted studies on SIRT1 KO and WT mice embryonic fibroblasts (MEFs). They found significantly elevated level of acetylation and phosphorylation of STAT3 in SIRT1 KO MEFs compared to WT ones. Whereas, the cell culture treatment with SIRT1 inhibitor (EX527) increased the level of acetylation and phosphorylation of STAT3 only in WT MEFs. These data indicate that STAT3 deacetylation is dependent on SIRT1.

### 2.3. Protein Inhibitor of Activated STAT (PIAS)

PIAS is a family consisting of four multifunctional proteins (PIAS1–4). PIAS proteins play an important role in the modulation of multiple signaling pathways through different molecular mechanisms [92,93]. The role of PIAS proteins in somatotropic axis has not been thoroughly investigated and their mechanism of action is based on the premises about the effect of PIAS exerted on STAT proteins [94,95].

The only available report about the role of PIAS in the somatotropic axis signaling is derived from the studies conducted on fish model [96]. According to Wong et al. [96], administration of the GH increased expression of PIAS1 mRNA in grass carp (*Ctenopharyngodon idella*), whereas PIAS1 overexpression induced inhibition of STAT5-mediated promoter activity. The results may indicate the existence of the negative feedback loop between somatotropic axis and PIAS1 protein in the grass carp.

**Figure 4:**
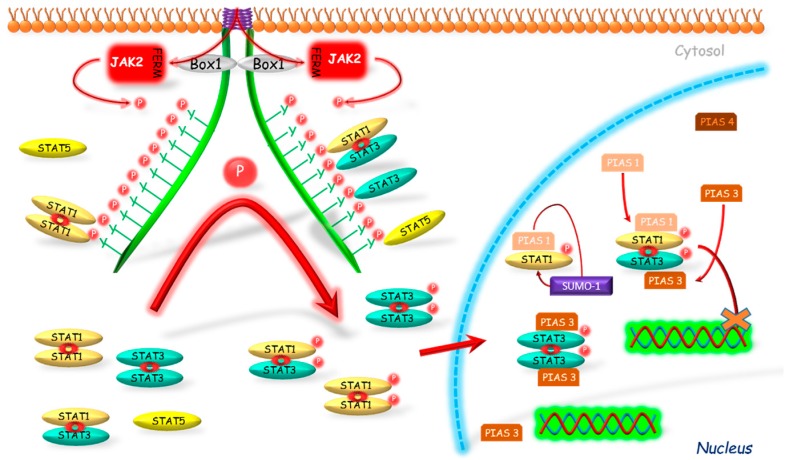
Connected with STAT’s DNA binding inhibition mechanism of PIAS action. PIAS proteins inhibit binding of phosphorylated STATs to their DNA promoter regions. Another mechanism is associated with a sumoylation of STATs which results in inhibition of activity of these proteins. JAK2—Janus kinase 2; FERM—N-terminal 4.1, Ezrin, Radixin, Moesin domain; Box 1—proline-rich domain; P—phosphorylation marker; PIAS—protein inhibitor of activated STAT; SUMO—E3-type small ubiquitin-like modifier; STAT—signal transducers and activators of transcription; cross—inhibition.

Liu et al. [97] stated that STAT protein may be modulated by PIAS proteins in varied ways. The authors indicated three reasons for such differentiation (Figure 4). Firstly, the interaction of PIASs with STATs may be type-dependent, e.g., PIAS1-STAT1 and PIAS3-STAT3. Secondly, the PIASs can inhibit STAT-induced gene expression by varied mechanisms: DNA binding inhibition (e.g., STAT1-PIAS1) or without DNA binding inhibition (e.g., STAT1-PIAS4). The third cause is the differentiated localization of PIASs proteins in tissues, e.g., in thymus, the presence of PIAS1 but not PIAS4 is observed.

The inhibitory effect of PIAS proteins on STAT DNA binding activity can be achieved by several possible mechanisms. Firstly, the binding of PIAS proteins to STAT dimer may mask the DNA binding domain of STAT. Secondly, PIAS proteins may bind to STATs and prevent their dimerization [98]. PIASs can also modulate the relocalization of transcriptional regulators to different subnuclear compartments. One of the mechanisms, which can participate in this process, is dependent on PIAS function as E3-type small ubiquitin-like modifiers (SUMO) ligases (Figure 4). The so-called sumoylation was identified as a mechanism for modulation of the effects of transcriptional factors, such as STAT1 [99]. Ungureanu et al. [99] demonstrated that STAT1 is modified by the SUMO-1 protein. The sumoylation of STAT1 was confirmed in both in vivo and in vitro studies in the one evolutionary conservative amino acid residue Lys703. It was stated that PIAS proteins strongly stimulate the sumoylation of STAT1, thus inhibiting its activity. Also PIAS3 may participate in the sumoylation as its two forms, a 68-kDa and a 85-kDa, were identified as correlating with the non-sumoylated and sumoylated form, respectively [100].

The studies on the PIAS action without DNA binding inhibition were conducted by Liu et al. [97]. The researchers demonstrated that PIAS4, localized mostly in the nucleus, has an inhibiting effect on STAT1 acting as the transcriptional corepressor. The authors presented several arguments to support their thesis. First, PIAS4 and STAT1 interaction is stated both in vitro and in vivo. As a second argument, the authors stated that PIAS4 can inhibit STAT1-induced activation; however, in contrast to PIAS1 and 3, it did not block the DNA binding activity of STAT1. Moreover, the authors also found the decreased inhibitory effect of PIAS4 on STAT1-induced genes when the expression of PIAS4 was high, which is so called “squelching” phenomenon typical for transcriptional coregulators. Another argument that authors mentioned is the presence of the LXXLL coregulatory signature motif, which is required for transrepressional activity of PIAS4 and which was identified in several nuclear corepressors.

Liu et al. [98] demonstrated that PIAS1 binds to STAT1 but not to STAT2 or 3, whereas PIAS1-STAT1 interaction requires STAT1 phosphorylation on Tyr701. It has also been shown that only PIAS1, from the whole PIAS family, is able to block STAT1 binding to DNA, and thus to inhibit gene expression induced by this protein. Liu et al. [101] in order to investigate PIAS1 physiological functions, created PIAS1 KO mice. Their results confirmed that PIAS1 is a physiological negative regulator of STAT1 and that PIAS1 disrupts binding of STAT1 with endogenous promoters of genes. Detailed investigations with use of microarray assay were performed and shown an unexpected PIAS1 role in the regulation of genes expression mediated by interferon (IFN)-γ or IFN-β. Functional studies results suggest that PIAS1 is an important factor in IFN-mediated response of innate immune system [101].

Chung et al. [102] conducted studies to identify STAT proteins able to interact with PIAS3. Their results excluded binding of STAT1 to PIAS3, but showed the interaction between PIAS3 and STAT3. In addition, researchers observed a complete inhibition of STAT3 homodimer and STAT1-STAT3 heterodimer binding to DNA by PIAS3. However, in the case of STAT1 homodimer this effect was not observed. Rycyzyn and Clevenger [103] conducted studies on the prolactin receptor and showed for the first time the interaction between STAT5 and PIAS3.

### 2.4. Phosphatases

#### 2.4.1. Protein Tyrosine Phosphatase (PTP-1B and PTP-H1)

**Figure 5:**
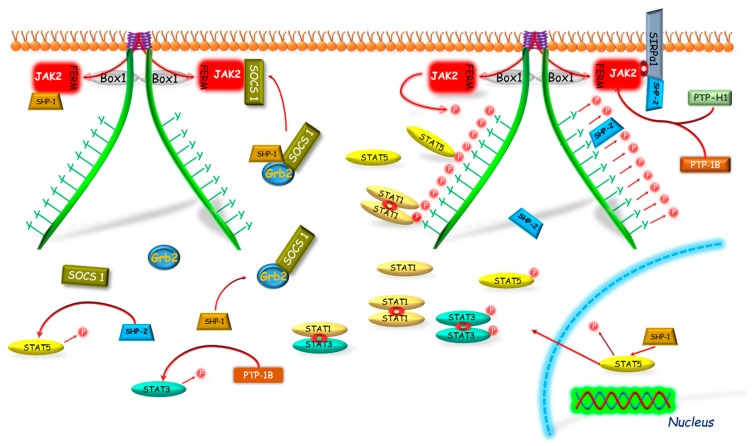
GHR-JAK2-STAT pathway inhibition mediated by PTP-1B, SHP-1, SHP2 and SIRPα1. PTP-1B dephosphorylates residual tyrosines of JAK2, GHR, STAT3 or STAT5. PTP-H1 dephosphorylates GHR acting through the JAK2 activity down-regulation. SHP-1 mechanism of action: direct dephosphorylation of JAK2; targeting of the SOCS1–Grb2 complex to JAK2; dephosphorylation of STAT5β in nucleus. SHP-2 mechanism of action: direct dephosphorylation of STAT, binding of SHP-2 to phosphotyrosines and dephosphorylation of GHR complex; binding of SHP-2 to JAK2 via SIRPα1. SIRPα1 after the GHR activation binds to JAK2 and creates binding site for SHP-2. Box 1—proline-rich domain; FERM—N-terminal 4.1, Ezrin, Radixin, Moesin domain; GHR—growth hormone receptor; Grb2—growth factor receptor-bound protein 2; JAK2—Janus kinase 2; P—phosphorylation marker; PTP—Protein tyrosine phosphatase; SHP—Src homology phosphatase; SOCS1—suppressor of cytokine signaling 1; STAT—signal transducer and activator of transcription.

The next factors regulating the somatotropic axis activity are the protein tyrosine phosphatases. Pasquali et al. [104] identified two PTPs involved in the GHR signaling: PTP-1B and PTP-H1. PTP-1B is broadly expressed in whole body tissues [105]. Gu et al. [106] examined the influence of PTP-1B on the signal transduction of the GH (Figure 5). The authors showed that in the MEFs isolated from PTP-1B KO mouse embryos the GH-dependent hyperphosphorylation of JAK2 and enhanced activation of STAT3 and STAT5 were observed. The authors also stated that PTP-1B restricts the ligand-dependent signaling by dephosphorylation of residual tyrosine of JAK2, but without excluding the dephosphorylation of another parts of the GH pathway such as GHR, STAT3 or STAT5. As a consequence of the overexpression of PTP-1B, the reduction of GH-induced expression of STAT5-dependent genes was observed. Moreover, the results of Gu et al. [106] indicate that the functional significance of PTP-1B in the acute response to GH varies depending on the nutritional status. Using the PTP-1B KO mice (hybrid of 129S/v and BALB/c backgrounds) the authors demonstrated that the lack of PTP-1B in well-nourished mice did not affect the GH-induced activation of JAK2, STAT3 nor STAT5 in the liver. However, during the starvation the authors stated significantly higher level of activation of these proteins in the PTP-1B KO mice compared to control group. In WT fasted mice the GH resistance state develops, which is manifested by disorders in somatotropic axis signal transduction at the GHR level, whereas in fasted PTP-1B KO mice this mechanism is impaired and, despite starvation conditions, GH resistance state does not developed.

Likewise other researchers conducted studies on the PTP-1B KO mice and observed significant growth disturbances. Escrivá et al. [107] stated that PTP-1B KO mice had higher body and liver weight at the 3rd day after birth. The same authors showed that PTP-1B KO mice were characterized by the increased STAT5β phosphorylation and, in consequence, the increased level of the IGF-1 in liver was stated. Also Owen et al. [108] planned a study to investigate the role of PTP-1B in GH signal transduction in the liver. This team conducted an experiment on mice with the local deletion of the gene coding PTP-1B protein (liver-specific PTP-1B^−/−^) and compared the impact of standard and high-fat diets. The body weight and fatness were similar in the females from the liver-specific PTP-1B^−/−^ and control groups. In animals fed standard diet, after 48 h of fasting the GH resistance state developed and an intraperitoneal GH administration led to stimulation of the JAK-STAT signaling pathway. At the same conditions the mutants showed significantly higher GH-induced phosphorylation of JAK2 and expression of the *SOCS3* gene. However, the STAT3, STAT5 and ERK1/2 phosphorylation and *SOCS2* gene expression were similar in both groups. Significantly higher level of mechanistic target of rapamycin (mTOR) phosphorylation was observed in mutants 5 min after the GH administration. Under the high-fat diet condition, the GH-induced phosphorylation of STAT5 was significantly higher in liver-specific PTP-1B^−/−^ mice, but did not change in the control group. The authors pointed out that the deletion of the *PTP-1B* gene in the liver leads to significant changes in the GH signal transduction as well as to the increased phosphorylation of JAK2 and STAT5, and expression of *SOCS3* gene.

Pasquali et al. [104] observed that PTP-H1 also exerts an inhibitory effect on the GHR signaling. The authors observed that this phosphatase, similarly to PTP-1B, dephosphorylated the GHR activated by ligand, whereas the effect on non-bound receptor was not stated. PTP-H1 probably acts through the JAK2 activity down-regulation (Figure 5). Pilecka et al. [109] confirmed the impact of PTP-H1 on the GHR signal transduction. The authors showed that male PTP-H1 KO mice were lighter than WT male mice; however, in females the difference was not statistically significant. They also observed that PTP-H1 KO mice of both genders had significantly higher expression of IGF-1 mRNA in liver and IGF-1 concentration in serum, and these differences were more pronounce in male mice.

#### 2.4.2. Src Homology 2 (SH2) Domain Containing Protein Tyrosine Phosphatase (SHP-1)

Src homology 2 (SH2) domain containing protein tyrosine phosphatase (SHP-1) is encoded by the *PTPN6* gene. Its presence was stated, inter alia, in the bone marrow, including the hematopoietic cells [110] and mesenchymal stem cells (MSC) [111]. In the nervous system the SHP-1 is present in astrocytes, hippocampal pyramidal cells, brain cortex [112] or oligodendrocytes [113]. SHP-1 affects the inhibition of the cytokine receptors signal transduction, tyrosine kinase receptors and immune system receptor complexes. Dephosphorylation of receptors and/or associated kinases has been described as a possible mechanism of SHP-1 effect [114]. Using the prolactin-dependent pre-T lymphoma cell line, Minoo et al. [114] demonstrated, based on prolactin receptor, that the inhibitory effect of SHP-1 is dependent on growth factor receptor-bound protein 2 (Grb2)-SOCS1 complex. The authors suggested that the inhibitory effect on the signal transduction of the cytokine receptors mediated by SHP-1 is due to the targeting of SOCS1 to JAK2 by this phosphatase, and that this may be the common mechanism in cytokine receptors. Moreover, it was stated also that under the stimulation of GH the SHP-1 binds directly to JAK2 [115]. Hackett et al. [115] stated also that in motheaten mice, which are deficient in SHP-1, prolonged activation of STATs and tyrosine phosphorylation of JAK2 was observed. Ram and Waxman [116], conducting research on rats and the CWSV-1 rat hepatocytes cell line, demonstrated that GH activates SHP-1 and induces its translocation into the nucleus, where SHP-1 binds to STAT5β, which participates in the termination of GH signal transduction in male rat liver.

#### 2.4.3. Src Homology 2 (SH2) Domain Containing Protein Tyrosine Phosphatase (SHP-2)

SHP-2 (Src homology phosphatase 2), also called tyrosine-protein phosphatase non-receptor type 11 (PTPN11), protein tyrosine phosphatase 1D (PTP-1D) or protein-tyrosine phosphatase 2C (PTP-2C), is an enzyme that is encoded in human by the *PTPN11* gene. SHP-2 is built of two chains, each of which contains three domains: one tyrosine phosphatase domain and two SH2 domains (C-SH2 and N-SH2) [117]. SHP-2 is widely distributed in signaling pathways of growth factors or cytokines [118], both as a positive regulator and inhibitor [117]. SHP-2 plays an important role in the regulation of GH-GHR signaling. Stofega et al. [78] investigated the role of SHP-2 in GH signal transduction by introducing the mutation in Tyr595 site of the GHR, which is the place of SHP-2 binding (Figure 5). The mutation resulted in the significant prolongation of the GHR phosphorylation after activation of this receptor. The two phosphorylated tyrosines at 595 site on each GHR monomer simultaneously bind to the N- and C-terminus of SH2 domains of SHP-2 resulting in activation of this phosphatase. Subsequently, the activated SHP-2 dephosphorylates associated GHR [78]. The results of the same team indicate that the mutation of the SHP-2 binding site also prolongs the JAK2 phosphorylation. SHP-2 binding to Tyr595 site of the GHR potentially dephosphorylates also the associated JAK2. Depending on the dephosphorylated tyrosine, the JAK2 dephosphorylation may deactivate or remove binding sites for specific signaling molecules. Aforementioned authors investigated the effect of mutation of SHP-2 binding site at GHR on the STAT5β tyrosine phosphorylation. The authors observed the significantly prolong tyrosine phosphorylation of the STAT5β and enhancement of STAT5β dependent activation of spi 2.1 reporter protein. Such effect was possible because of repeated replication of the STAT5β phosphorylation cycle by JAK2. As it was showed by Yu et al. [119], the SHP-2 but not SHP-1 directly dephosphorylates STAT5 and maintains a basal steady state level of this protein activity.

There are various mechanisms regulating SHP-2 catalytic activity. The first one is connected with dual activity of N-SH2 domain [117]. The N-SH2 domain either binds phosphatase domain damaging SHP-2 phosphopeptide binding cleft or binds phosphopeptide ligand inactivating its phosphatase domain recognizing area. Therefore, the authors stated that the N-SH2 domain is a conformational switch that simultaneously inhibits and activates the activity of SHP-2 [117]. In turn, the C-SH2 domain does not interact with the N-SH2 and phosphatase domains, and therefore it is considered that C-SH2 domain does not exert great impact on the SHP-2 catalytic activity. However, the binding of C-SH2 to a phosphotyrosine-containing ligand may enhance local ligand concentration and increase its availability for the N-SH2 domain [120]. Another mechanism that affects the catalytic activity of SHP-2 is its dimerization. Nardozza et al. [121] have shown that SHP-2 dimers are 3 times less active than the monomers and that this mechanism can be modulated by the redox state of a cell. SHP-2 may dimerize in vivo, which participates in maintaining a stable SHP-2 pool in the inactive conformation in non-stimulated cells. The results of the authors indicated that at least 15% of SHP-2 in vivo occur in the dimer form and the dimer/monomer ratio constantly changes, which inversely correlated with the activity of the mitogen-activated protein kinases (MAPK) pathway. Nardozza et al. [121] found that the SHP-2 was able to dimerize in conditions of growth factor and nutrient deficiency, while incubation with the epidermal growth factor (EGF) caused dissociation of the dimers.

The important role of the SHP-2 in the regulation of the GH signal transduction is confirmed by two syndromes, Noonan (NS) and Leopard (LS) syndrome, which are primarily caused by mutation in the *PTPN11* gene [122,123]. Many symptoms in both these diseases are similar, such as low growth, facial dimorphism or skeletal abnormalities [122,124,125]. The NS results from nonsense mutations of the *PTPN11* gene, which affects the proteins involved in the interaction of N-SH2 and PTP domains resulting in chronic stabilization of the active conformation of the SHP-2 molecule, and thus the gain-of- inhibitory function [124]. The LS results from the decreased catalytic activity of SHP-2 and the lack of agonistic effects of SHP-2 on ERK/MAPK signaling pathway [126].

### 2.5. Signal Regulatory Proteins (SIRPs)

Signal regulatory proteins (SIRPs) are glycoproteins characterized by the presence of immunoglobulin-like domains located extracellularly. In contrast to SIRPβ subgroup, SIRPα1 has a cytoplasmic domain containing four immunoreceptor tyrosine-based motifs (ITIM) and a proline rich region [127]. By the presence of Tyr located in cytoplasmic domain, SIRPs proteins can bind to the SH2 domains of the SHP-2 protein [128,129] (Figure 5). It was found that SIRPα1 is involved in signal transduction of cytokine receptors, including GHR [130]. In response to GH, SIRPα1 binds to JAK2, which stimulates SIRPα1 phosphorylation and creates binding site for SHP-2. Further studies of Stofega et al. [78] showed that the binding of SIRPα1 to JAK2 does not require the presence of neither phosphotyrosine in SIRPα1 and JAK2 nor the proline rich region in SIRPα1. However after the deletion of the 30-C-terminus amino acid fragment from SIRPα1 molecule, which contains the proline-rich region and Tyr495, the tyrosine phosphorylation of SIRPα1 by JAK2 and its binding to SHP-2 are inhibited. The expression of SIRPα1 has an inhibitory effect on the GH-induced phosphorylation of JAK2, which suggests its impact on signal transduction of the GHR-JAK2 pathway. Moreover, Stofega et al. [78] demonstrated that SIRPα1 decreased also, GH-induced phosphorylation activity of ERK1/2, STAT3 and STAT5. These results suggest that SIRPα1 plays a role as a negative regulator of GH signal transduction. However, SIRPα1 role is supposed to be limited to the creation of binding sites for SHP-2, which increases their phosphatase activity (Figure 5).

## 3. Concluding Remarks

Considering the pleiotropic role of the somatotropic axis in the whole organism, studies aimed at understanding its regulatory mechanisms seem very important. In this review, the several physiological inhibitors of the GHR-JAK2-STAT pathway, which act through numerous different mechanisms (e.g., competitive binding, dephosphorylation, ubiquitination, sumoylation), have been presented. The understanding of the physiological role of the GHR-JAK2-STAT pathway may have a profound importance for current medicine. It is well known that GHR regulates many cellular pathways involved in survival, proliferation, metastasis, epithelial to mesenchymal transition, apoptosis and cell cycle, but its hyperactivation may increase the risk of cancer in general [131]. GHR activates many oncogenic pathways, such as SRC, Ras/ERK, PI3-kinase and NF-κB. It was stated that also STAT3 and, in particular, STAT5 activated by GHR may participate in the ontogenesis and cancer progression (widely described by Chhabra et al. [132]. Overexpression of aforementioned STAT3 and STAT5 was also observed in a wide range of cancers and may contribute in tumor progression and cell survival [133,134]. Therefore, broadening knowledge about the inhibitors of the GHR-JAK2-STAT pathway may be important in the development of new anti-cancer therapy. Although these inhibitors do not probably replace the conventional drugs used in the treatment of cancer, their usage may support the conventional therapy allowing to reduce the doses of currently used drags, which could be useful in the reduction of the negative impact of standard anti-cancer treatment. Such kinds of therapy have been already tested among others by Vendrely et al. [135]. Authors investigated the impact of mix of resveratrol (which is commonly used as a stimulator of SIRT1 expression), capsaicin, piceatannol and sulphoraphane in varied combinations with analog of gemcitabine, which is used in the systemic chemotherapy, on apoptosis of pancreatic adenocarcinoma cell lines. The studies have shown that mix of bioactive food components potentiated chemotherapy and maintained its full efficiency when gemcitabine dose was lowered by 1/3rd in adenocarcinoma therapy.

Besides the modulation of the equilibrium between activity and inhibition of the described GHR-JAK2-STAT pathway inhibitors, the medical approach directed to new synthetic inhibitors of this pathway’s main components (JAK2 and STATs) is of the wide medical interest. Many of JAK’s and STAT’s inhibitors have being currently tested in various conditions from asthma to malignancies, myeloproliferative diseases or autoimmune conditions. The emergence of a new class of therapeutics targeted at the JAKs (Ruxolitinib, Tofacitinib, Oclacitinib), which have been already approved by the Food and Drug Administration (FDA), is of a great importance in modern animal and human medicine. The next challenge of the pharmaceutical industry is the development of the STAT inhibitors with clinical relevance [136]. Therefore better understanding of the role and therapeutic potential of the GHR-JAK2-STAT pathway inhibitors may certainly contribute to the development of new more effective and safer therapies of a wide variety of diseases.
